# Toxicities associated with lymphoma-targeting bispecific antibodies—a review

**DOI:** 10.3389/fmed.2025.1582975

**Published:** 2025-07-02

**Authors:** Christopher Doig, Costas Kleanthes Yannakou

**Affiliations:** ^1^Department of Molecular Oncology and Cancer Immunology, Epworth HealthCare, Melbourne, VIC, Australia; ^2^Department of Clinical Pathology, The University of Melbourne, Parkville, VIC, Australia

**Keywords:** BsAb, bispecific antibody, CRS, cytokine release syndrome, ICANS, immune effector cell-associated neurotoxicity syndrome, CD20, lymphoma

## Abstract

Bispecific antibodies (bsAbs) are an emerging class of directed immunotherapies with established uses in certain hematological malignancies as well as an emerging role in the treatment of solid organ malignancy. These molecules are able to juxtapose T cells (in most cases) with target tumor cells, forming an immunological synapse. bsAbs are under extensive investigation in the treatment of B-cell non-Hodgkin lymphomas, with encouraging results in both the monotherapy and combination therapy settings. In this review we summarize the key toxicities associated with the use of lymphoma-targeting bsAbs: cytokine release syndrome, immune effector cell associated neurotoxicity syndrome, cytopenias, infections and immunosuppression as well as tumor lysis syndrome. While the toxicities are not insignificant, they are typically manageable and justifiable given the unmet medical need, especially in the case of relapsed or refractory disease.

## Introduction

The treatment of relapsed or refractory B-cell non-Hodgkin lymphoma (NHL) is a challenging but evolving field. The emergence of therapies which harness the immunological response, such as bispecific T- and NK-cell engagers as well as chimeric antigen receptor (CAR) T cells, has opened a new therapeutic frontier in the management of NHL. Nevertheless, the use of CAR-T cells has been limited by practical considerations such as availability, cost and toxicities.

Bispecific antibodies (bsAbs) are an emerging class of directed immunotherapies with established uses in certain hematological malignancies as well as an emerging role in the treatment of solid organ malignancy. Since the initial FDA approval of blinatumomab for use in acute lymphoblastic leukemia in 2014 (1), a number of agents have shown promise in clinical trials for the treatment of B-cell NHLs.

bsAbs are manufactured antibodies with dual specificity: for a target antigen on the tumor cell (e.g., CD19 or CD20) and for a target antigen on an effector cell (e.g., CD3 for T cells). These molecules are able to juxtapose T cells (in most cases) with target tumor cells, forming an immunological synapse while bypassing the need for the major histocompatibility complex (MHC)—T-cell receptor (TCR) interaction (2).

While the efficacy of these agents is outside the scope of this review, a number of safety concerns have been raised as class effects. In particular, the adverse effects of cytokine release syndrome (CRS) and immune effector cell-associated neurotoxicity syndrome (ICANS) are seen with both bsAb and CAR-T cell therapies. Further safety concerns include cytopenias and infectious complications.

This review will aim to summarize and characterize the adverse effects associated with bsAb therapy in the treatment of lymphomas.

## bsAbs approved for use in B-cell lymphoma

Modifications to the structure of bsAbs have advanced their safety, efficacy, and pharmacokinetic properties. Blinatumomab is composed of single chain variable fragments (scFVs) joined by a linking domain (3). The structure of blinatumomab results in rapid renal clearance, requiring it to be administered as a continuous IV infusion. Other bsAbs currently used in NHL (glofitamab, mosunetuzumab, epcoritamab, and odronextamab) have structures more similar to that of IgG and so have half-lives closer to that of endogenous IgG (4–7). Odronextamab is different amongst these for being of IgG4 subtype, while the remainder are IgG1.

The challenge of ensuring correct heterodimerisation during the manufacturing process has led to the use of several strategies. The use of the knobs-into-holes method (glofitamab, mosunetuzumab) (8), matched CH3 mutations (epcoritamab) (8) and point mutations ablating protein A binding (odronextamab) (9) allow for purification and pairing. All CD3xCD20 bsAbs in use have non-functional Fc regions. While there are insufficient data to draw definitive conclusions about the clinical implications of bsAb Fc domain functionality, a functional Fc receptor should be capable of signaling to antigen-presenting cells and so may induce antibody-dependent cellular cytotoxicity, however, this may also increase the potential for toxicity (10).

A further modification has been the incorporation of an additional anti-CD20 Fab moiety. This is designed to enhance avidity for B cells, and differentiates glofitamab from other bsAbs in use. A comparison of structural differences between CD3xCD20 bsAbs is provided in Table 1.

**TABLE 1:** Structural comparison of CD3xCD20 bsAbs.

Drug	Glofitamab	Mosunetuzumab	Epcoritamab	Odronextamab
Structure	2:1 humanized IgG1	Humanized IgG1	IgG1-like	Humanized IgG4
	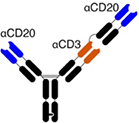	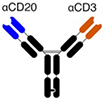	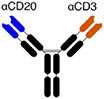	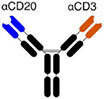
Fc segment	Non-functional	Non-functional	Non-functional	Non-functional
Heavy chain pairing method	Knob-into-hole	Knob-into-hole	Matched point mutations in CH3 domains	Point mutations ablating protein A binding
Administration	IV	IV	Subcutaneous	IV
Target dose (RP2D)	30 mg 3-weekly (11)	60 mg 3-weekly (12)	48 mg weekly (13)	80–160 mg weekly (14)

Efficacy outcomes from major monotherapy studies of bsAbs are summarized in Table 2.

**TABLE 2:** Efficacy outcomes of CD3xCD20 bsAb monotherapy studies.

Drug	Study	Disease	*n* =	ORR	CR	Median OS	Median PFS	Median follow-up (range)
Glofitamab	Rentsch et al. (15)	DLBCL	9	67%	44%	N.R. (ND)	161 d (ND)	246 d (15-482)
	Birtas Atesoglu et al. (16)	DLBCL	46	37%	21%	8.8 m (95% CI 4.85–12.89)	3.3 m (95% CI 2.34–4.35)	5.7 m (0.3–14.19)
	Sesques et al. (17)	B-NHL	63	ND	DLBCL: 36.4% Other*: 52.6%	DLBCL: 17.6 m (90% CI 8.3–19.7) Other*: NR (90% CI 4.6–NR)	DLBCL: 4.9 m (95% CI 2.6–19.7) Other*: 4.1m (95% CI 1.4–NR)	DLBCL: 9.7 m (95% CI 8.1–11.8) Other*: ND
	Hutchings et al.^†^ (18)	B-NHL	35	65.7%	57.1%	ND	ND	ND
	Dickinson et al. (19)	DLBCL	154	52%	39%	ND	4.9 m (95% CI 3.4–8.1)	12.6 m (0.1–22.1)
	Phillips et al. (20)	MCL	60	85%	78.3%	29.9 m (95% CI 17.0–NE)	16.8 m (95% CI 8.9–21.6)	19.6 m (0–39)
	Haynes et al. (21)	NHL	142	61%	27%	ND	ND	6.1 m (IQR 2.5–9.1)
	Brooks et al. (22)	LBCL	70	53%	25%	7.2 m (95% CI 6.1–NR)	2.7 m (95% CI 2.0–3.9)	5 m (ND)
	Tong et al. (23)	DLBCL/HGBL	28	62.5%	25%	NR (ND)	NR (ND)	3 m (ND)
Mosunetuzumab	Budde et al. (12)	FL	90	80%	60%	NR (NR – NR)	17.9 m (95% CI 10.1–NR)	18.3 m (IQR 13.9–23.3)
	Bartlett et al. (24)	DLBCL	88	42%	23.9%	11.5 m (95% CI 9.0–16.4)	3.2 m (95% CI 2.2–5.3)	10.1 m (ND)
Epcoritamab	Thieblemont et al. (25)	LBCL	157	63.1%	38.9%	18.5 m (95% CI 11.7–27.7)	4.4 m (95% CI 3.0–8.8)	25.1 m (95% CI 24.0–26.0)
	Izutsu et al. (26)	B-NHL	9	55.6%	44.4%	ND	ND	14.9 (ND)
	Izutsu et al. (26)	DLBCL	36	55.6%	44.4%	NR (95% CI 8.1–NR)	4.1 m (95% CI 0.5–NR)	8.4 m (95% CI 6.5–11.2)
	Haynes et al. (21)	NHL	38	ND	26%	ND	ND	6.5 m (IQR 1.5 – 8.5)
	Brooks et al. (22)	LBCL	139	49%	23%	7.2 m (95% CI 6.1–NR)	2.7 m (95% CI 2.0–3.9)	5 m (ND)
Odronextamab	Bannerji et al. (14)	B-NHL	145	51%	37%	ND	FL: 17.1 m (95% CI 7.5–NE) DLBCL, no prior CAR-T: 11.5 m (95% CI 0.5–NE) DLBCL, prior CAR-T: 2 m (95% CI 0.9–5.3)	4.2 m (IQR 1.5–11.5)
	Ayyappan et al. (27)	DLBCL	141	52%	31%	ND	ND	26.2 m (ND)
	Kim et al. (28)	FL	128	80%	73%	NR (ND)	20.7 m (ND)	20.1 (ND)

ND, no data. NR, not reached. *Other, follicular lymphoma, mantle cell lymphoma, transformed follicular lymphoma, transformed marginal zone lymphoma, primary mediastinal B cell lymphoma, transformed Waldenstrom macroglobulinaemia.

^†^Safety-evaluable (RP2D) cohort.

### Cytokine release syndrome

CRS has been a long-recognized complication of immune effector cell engaging therapies (bsAbs and CAR-T cells) in which unintended, hyperinflammatory responses result from exposure to therapy. CRS may occur soon after drug administration but may also be delayed by hours or even days to weeks (29). The clinical presentation is primarily characterized by fever, with or without other features such as hypoxia, and hypotension. CRS can progress to multiorgan failure and can range in severity from mild to life-threatening (30). The CRS associated with bsAbs typically occurs relatively shortly after the time of infusion, as opposed to with CAR-T cells where this may present later, at the time of CAR-T cell expansion (31).

CRS is caused by the excessive release of inflammatory cytokines from hyperactivation of both lymphoid and myeloid cellular components of the immune system. Key mediators include interleukins (IL)-1 and -6, tumor necrosis factor (TNF)-α, and interferon (IFN)-γ, however, numerous other cytokines have also been implicated (32). IL-1 and IL-6 are the targets of therapeutic intervention with treatments such as anakinra and tocilizumab, respectively.

CRS is also a recognized complication of a number of immunotherapies that do not directly engage the immune effector cell such as the monoclonal antibodies e.g., rituximab (33) and alemtuzumab (34), as well as with checkpoint inhibitors (35). There is significant overlap in clinical presentation and pathophysiology between CRS and other hyperinflammatory conditions such as haemophagocytic lymphohistiocytosis (36), engraftment syndrome (37), and immune reconstitution inflammatory syndrome (38) as well as certain infections including SARS-CoV-19 (39).

Several clinicopathological scores have been developed for the assessment and grading of CRS (30, 40, 41). Earlier scoring systems, including previous editions of the Common Terminology Criteria for Adverse Events (CTCAE), used criteria incorporating cessation of infusion, however, the 2017 CTCAE update removed this criterion due to an increased appreciation of delayed CRS. While this grading system is commonly used for CRS associated with bsAbs, a separate system proposed by the American Society for Transplantation and Cellular Therapies is used for grading CAR-T-associated CRS (41).

Management of CRS associated with bsAbs depends on severity. Supportive measures are utilized to some degree in all cases, depending on grade, and may include antipyretics, intravenous fluids, supplemental oxygen and vasopressor support. In cases of grade 2 or higher CRS or prolonged fevers, treatment with tocilizumab (IL-6 blockade) with or without dexamethasone is warranted. Refractory cases can be treated with alternative IL-6 blockade (siltuximab) or IL-1 blockade (anakinra) (42). Furthermore, given the undifferentiated nature of fever, concurrent empiric management for sepsis is usually warranted.

In order to reduce the incidence and severity of CRS, the practice of “step-up dosing” whereby increasing doses are administered over time has become ubiquitous in the administration of bsAbs (43). The risk of developing CRS varies between cycles, with odronextamab and glofitamab studies demonstrating the highest incidence of CRS with initial dosing, while epcoritamab and mosunetuzumab having higher incidence following full-dose treatment (14, 44).

While comparative studies are lacking, the use of subcutaneous dosing of mosunetuzumab has shown promisingly low rates of CRS in a recent study, suggesting that the route of administration may be of relevance (45). In addition, it has been demonstrated that premedication with dexamethasone may reduce the risk of CRS when compared with other corticosteroids such as prednisolone or methylprednisolone (46).

Nine studies have investigated the use of glofitamab as monotherapy to date, with a total of 607 patients included (15–23). All of these studies investigated relapsed or refractory lymphoma, either diffuse large B-cell lymphoma, mantle cell lymphoma, or B-cell non-Hodgkin lymphoma more broadly. All studies used pre-treatment obinutuzumab, and used a “step-up dosing” schedule for glofitamab. Of note, Phillips et al. (20) compared two fixed doses of obinutuzumab before glofitamab. The 2,000 mg obinutuzumab dose resulted in a lower incidence and severity of CRS compared with the 1,000 mg dose (20).

Rates of CRS varied between studies, ranging from 14.3 (17) to 70% (20), noting some heterogeneity in the definition of CRS. In all studies grade 1–2 CRS made up the majority of overall CRS. In comparison to other studies, Birtas Atesoglu et al. (16) demonstrated low overall rates of CRS (27.9%), but higher proportions of higher grade CRS (Gr 1–2 16.6%, Gr 3–4 9.3% and Gr 5 2%). This real-world study was also notable for higher prior lines of therapy (median 4 vs. median 3 in other studies), and younger median age (54 yrs vs. 60–68 yrs) suggesting that patient selection differences may be of relevance.

Notably, two glofitamab studies investigated only patients who had received prior CAR-T cell therapy (15, 17), while one did not report this data (16) and in three studies, 60, 33 and 2.9% of patients had received prior CAR-T cells (18, 19, 21). In those with prior CAR-T exposure, CRS was seen in 22% (15) and 14.3% (17), while in Dickinson et al. (19) (33% prior exposure), CRS was seen in 63% of patients. In comparison, in Hutchings et al. (18) (2.9% prior exposure), CRS occurred in 57.9% of patients.

Mosunetuzumab has been studied in two monotherapy trials (12, 24) involving a total of 178 patients. These studies differed significantly by disease type studied, with Budde et al. (12) limited to follicular lymphoma and Bartlett et al. (24) limited to DLBCL. Nevertheless, both studies used comparable methodologies including corticosteroid pre-treatment and step-up dosing. Both studies were of relapsed disease with median 3 prior lines of therapy for both. CRS was reported in 44% in the FL study and 26.1% in the DLBCL study, with comparable rates of grades 1–2 CRS. A total of 29.5% of DLBCL patients, compared with 3% of FL patients had received prior CAR-T cell therapy.

Of note, Budde et al. (12) reported a single death due to haemophagocytic lymphohistiocytosis (HLH) on day 8 of treatment. While immune effector cell associated haemophagocytic syndrome (IEC-HS) is a recognized risk of CAR-T cell therapy, the risks of a similar syndrome with T cell-engaging bsAb therapy has not been established (47).

Four studies have investigated epcoritamab monotherapy (21, 22, 25, 26) in 179 patients. Thieblemont et al. (25) investigated R/R LBCL and demonstrated CRS in 51%, with 3.2% experiencing CRS grades 3–5. Izutsu et al. (26) incorporated a small (*n* = 9) dose escalation cohort involving patients with B-NHL, followed by a DLBCL-only expansion cohort, in which 83.3% experienced CRS overall, with 8.4% grades 3–5. Prior CAR-T had been used in 38.9% in Thieblemont et al. (25), and in 0% of the DLBCL-only cohort of Izutsu et al. (26).

Trials of odronextamab as monotherapy are ongoing, with encouraging and comparable results to other bsAbs. Preliminary analysis of the ELM-1 study by Bannerji et al. (14), which included 145 patients with mixed histology B-NHL, demonstrated 61% overall CRS rates (7% grades 3–5). Analysis of the DLBCL cohort of the ELM-2 study had similar results, with 55% CRS overall, and 1% grade 3–5 (27). Analysis of the FL cohort demonstrated 56% CRS overall, with 1.7% grade 3–5 (28).

### Immune effector cell associated neurotoxicity syndrome

Immune effector cell associated neurotoxicity syndrome (ICANS) is another complication of immune effector cell engaging therapies which is being increasingly encountered with the more widespread use of CAR-T cell therapies. While neurological toxicities have been reported in association with other immunotherapies (48–50), ICANS itself is a specific complication of immune effector cell engaging therapies, i.e., bsAbs and CAR-T cell therapies. ICANS that occurs in the setting of CAR T-cell therapies is in general more frequent and severe than that related to bsAbs (51).

Understanding the pathophysiology of ICANS in the specific context of CD3xCD20 bsAb use is an evolving field. One proposed mechanism is that IL-1 mediates disruption of the blood-brain-barrier (BBB) through bystander monocyte activation, and that subsequent translocation of T cells across the BBB leads to inflammation within the central nervous system (CNS) (52). Additional mechanisms of neurotoxicity have been proposed, implicating other myeloid cells and cytokines, as well as endothelial activation (53). It is likely that a class effect is present, as all CD3xCD20 bsAb monotherapy studies have reported neurotoxicity, albeit at much lower rates and severities than with CAR-T cells and CD3xCD19 bsAbs (54). It has been hypothesized that this difference is at least in part due to CD19 expression on pericytes in the BBB which do not express CD20 (55).

The clinical features of ICANS are varied and range from dysgraphia, inattention and expressive aphasia to seizures, elevated intracranial pressure, reduced conscious state and focal motor findings. The initial onset of ICANS can be subtle, thus frequent monitoring and a high index of clinical suspicion is required. Grading of ICANS is based on the American Society for Transplantation and Cellular Therapy (ASTCT) consensus grading score, which incorporates the Immune Effector Cell-Associated Encephalopathy (ICE) score (41) that was developed in the context of CAR-T cell therapy but that is commonly applied in the context of bsAbs. The ICE score is a 10-point scoring system which assesses patients for features of encephalopathy including orientation, naming of objects, ability to follow commands, writing, and attention. Other features assessed in the ASTCT grading include overall level of consciousness, seizures, motor findings and elevated intracranial pressure/cerebral oedema.

Treatment of ICANS is dependent on severity. In the case of bsAbs, pausing or discontinuing the infusion when possible (guided by severity) to allow for the resolution of ICANS features is an important aspect of management. The mainstay of treatment for ICANS is with glucocorticoids that penetrate the CNS such as dexamethasone and methylprednisolone. Due to tocilizumab’s limited CNS penetration it does not have a role in ICANS treatment, excepting when there is concurrent CRS (56). IL-1 blockade with anakinra has shown activity in severe, steroid-refractory ICANS in CAR-T trials and can be used in similar circumstances on a case-by-case basis in the context of bsAbs (57).

The incidence of ICANS-like events in CD3xCD20 bsAb trials has been difficult to establish given the significant heterogeneity in reporting. Nevertheless, ICANS appears to be a rare phenomenon in the setting of bsAbs, and overall appears to be predominantly of grade 1–2 severity.

Glofitamab monotherapy studies have all reported on neurological toxicity, rates of which have ranged from 0 to 8% overall, with grade 3–5 toxicity of 3% in the largest study (19). ICANS/neurological toxicity in mosunetuzumab has been reported as 2.2–3%, without any reported Gr > 2 toxicity. Epcoritamab monotherapy studies show ICANS of 0–10%, with one study (25) reporting one case of fatal ICANS. The three studies investigating odronextamab demonstrated ICANS/ICANS-like events in 0, 1.7, and 12% of cases, with Bannerji et al. (14) reporting 3% rate of Gr 3–5 ICANS-like events. It is important to note that the definition of neurotoxicity is heterogeneous between studies, with a minority of studies referring to the ASTCT definition.

Overall, there appears to be a modest correlation between the reported rates of CRS and ICANS. This is consistent with CAR-T data in which the development of ICANS is typically preceded by CRS (58). Despite it being challenging to establish the rate of ICANS-like features in the setting of different bsAbs, these appear to be low. There is also significant overlap between the treatment of CRS (which is more common) and ICANS, which may further obscure the true rate of neurological toxicity. Rates of CRS and ICANS are summarised in Table 3.

**TABLE 3:** CRS and ICANS incidence.

Drug	Study	Disease	*n* =	Prior CAR-T	CRS, all grade	CRS, Gr 1–2	CRS, Gr 3–5	ICANS, all grade	ICANS, Gr 1–2	ICANS, Gr 3–5
Glofitamab	Rentsch et al. (15)	DLBCL	9	100%	22%	22%	0%	0%^#^	0%	0%
	Birtas Atesoglu et al. (16)	DLBCL	46	ND	27.9%	16.6%	11.3%	3%^#^	3%	0%
	Sesques et al. (17)	B-NHL	63	100%	14.3%	14.3%	0%	3.2%^#^	3.2%	0%
	Hutchings et al.^†^ (18)	B-NHL	35	2.9%	37.1%	31.4%	5.7%	5.7%*	5.7%	0%
	Dickinson et al. (19)	DLBCL	154	33%	63%	59%	4%	8%*	5%	3%
	Phillips et al. (20)	MCL	60	3.3%	70%	58.4%	11.6%	11.7%^∧^	11.6%	0%
	Haynes et al. (21)	NHL	142	60%	33%	29%	4%	4%	3%	1%
	Brooks et al. (22)	LBCL	70	ND	28.6%	28.6%	0%	7.1%	5.7%	1.4%
	Tong et al. (23)	DLBCL/HGBL	28	21.4%	39.3%	32.2&	7.1%	ND	ND	3.6%
Mosunetuzumab	Budde et al. (12)	FL	90	3%	44**%**	42%	2%	3%^∧^	3%	0%
	Bartlett et al. (24)	DLBCL	88	29.5%	26.1%	23.8%	2.3%	2.2%*	2.2%	0%
Epcoritamab	Thieblemont et al. (25)	LBCL	157	38.9%	51%	47.8%	3.2%	6.4%^∧^	5.8%	0.6%
	Izutsu et al. (26)	B-NHL	9	22.2%	88.9%	77.8%	11.1%	0%^∧^	0%	0%
	Izutsu et al. (26)	DLBCL	36	0%	83.3%	75%	8.4%	2.8%^∧^	2.8%	0%
	Haynes et al. (21)	NHL	38	ND	33%	33%	0%	10%	7%	3%
	Brooks et al. (22)	LBCL	139	ND	51.1%	44.6%	6.5%	13.7%	10.1%	3.6%
Odronextamab	Bannerji et al. (14)	B-NHL	145	29%	61%	54%	7%	12%*	9%	3%
	Ayyappan et al. (27)	DLBCL	141	ND	55%	54%	1%	0%^∧^	0%	0%
	Kim et al. (28)	FL	128	ND	56%	54.3%	1.7%	0.8%	0.8%	0%

ND, no data.

^†^Safety-evaluable (RP2D) cohort. ^∧^ASTCT or investigator defined as ICANS. ^#^Neurotoxicity, unknown grading system. *CTCAE terms consistent with ICANS/ICANS-like events/potentially consistent with ICANS.

### Cytopenias

Cytopenias are frequently observed complications of bsAb therapy. The mechanisms by which these emerge are not well-established. Incidence is summarised in Table 4.

**TABLE 4:** Incidence of cytopenias.

Drug	Study	Disease	*n* =	Anemia, all grade	Anemia, Gr 3–5	Thromboc-ytopenia, all grade	Thromboc-ytopenia, Gr 3–5	Neutro-penia, all grade	Neutro-penia, Gr 3–5
Glofitamab	Rentsch et al. (15)	DLBCL	9	11%	11%	11%	11%	33%	22%
	Birtas Atesoglu et al. (16)	DLBCL	46	38.1%	19%	28.6%	19%	41.5%	23%
	Sesques et al. (17)	B-NHL	63	ND	11.1%	ND	11.1%	ND	33.3%
	Hutchings et al.^†^ (18)	B-NHL	35	0%	0%	ND	8.6%	ND	25.7%
	Dickinson et al. (19)	DLBCL	154	31%	6%	25%	8%	38%	27%
	Phillips et al. (20)	MCL	60	26.7%	11.7%	16.7%	10%	38.3%	23.3%
	Haynes et al. (21)	NHL	142	ND	ND	ND	ND	ND	22%
	Brooks et al. (22)	LBCL	70	ND	ND	ND	ND	ND	ND
	Tong et al. (23)	DLBCL/HGBL	28	ND	ND	ND	ND	ND	ND
Mosunetuzumab	Budde et al. (12)	FL	90	13%	8%	10%	4%	29%	26%
	Bartlett et al. (24)	DLBCL	88	17%	9.1%	ND	ND	27.3%	21.6%
Epcoritamab	Thieblemont et al. (25)	LBCL	157	21%	21.1%	12.1%	5.1%	23.6%	16.6%
	Izutsu et al. (26)	B-NHL	9	ND	ND	ND	ND	ND	ND
	Izutsu et al. (26)	DLBCL	36	11.1%	8.3%	22.2%	13.9%	38.9%	38.9%
	Haynes et al. (21)	NHL	38	ND	ND	ND	ND	ND	ND
	Brooks et al. (22)	LBCL	139	ND	ND	ND	ND	ND	ND
Odronextamab	Bannerji et al. (14)	B-NHL	145	28%	25%	28%	14%	25%	19%
	Ayyappan et al. (27)	DLBCL	141	43%	ND	ND	ND	ND	ND
	Kim et al. (28)	FL	128	33.6%	12%	ND	ND	39.1%	32%

^†^Safety-evaluable (RP2D) cohort. ND, no data.

The management of cytopenias is supportive and may include the use of transfusions, granulocyte colony stimulating factors, and thrombopoietin mimetics. The use of stored, autologous haematopoietic stem cell infusions has shown promise in patients with prolonged cytopenias following CAR-T cell therapy and may represent a viable option in selected patients following bsAb treatment (47). Temporarily withholding therapy can be considered, however, this risks losing disease control. It is also important to exclude other causes of cytopenias such as progressive disease.

### Infections and immunosuppression

Infectious complications have been variably noted across bsAb monotherapy studies. Ten studies reported overall infection rates, including all the CD3xCD20 bsAbs discussed above (11–15, 19–21, 26, 28). Budde et al. (12) study of mosunetuzumab in follicular lymphoma demonstrated the lowest rates of infection overall (20%), with higher rates (38–79.7%) in the other trials. Of note, febrile neutropenia was relatively rare, with rates of 0–5.7% in the 5 trials which reported this complication. Due to the non-uniform nature of reporting amongst studies, comparisons between different types of infections are challenging to interpret. COVID-19 was the most frequently reported individual infection, noting that these studies were primarily recruiting during the global pandemic. Furthermore, the use of immunosuppressive medications such as tocilizumab and corticosteroids in the treatment of CRS and/or ICANS are likely to increase infection risk. Given the overlapping clinical features of infection and CRS, establishing the contribution of immunosuppressive medications to infectious risk is difficult, however, in the context of other inflammatory disorders this relationship is well-established (59, 60).

Other indicators of immunosuppression are less well documented. In particular, the rates of acquired hypogammaglobulinemia have not been reported in the relevant trials, although this is an increasingly recognized consequence of CD3xBCMA bsAbs used in multiple myeloma (61). While no guidelines currently exist for replacement of immunoglobulins in this context, we suggest using intravenous immunoglobulin as primary or secondary prophylaxis as per institutional policies for the treatment of secondary hypogammaglobulinaemia.

In addition, continuous bsAb therapy has been suggested to result in T-cell exhaustion. One study has demonstrating decreased T cell cytotoxicity, decreased production of proinflammatory cytokines and increased expression of T-cell exhaustion markers following continuous blinatumomab (CD3xCD19 bsAb) administration in B-ALL using an in vitro model (62). The impact of fixed-duration versus continuous therapy with bsAbs on long-term infection risk is uncertain but remains an area for future study.

### Tumor lysis syndrome

Tumor lysis syndrome (TLS) is a recognized complication of bsAb therapy. TLS rates of 0–11% have been reported in the few studies that have published this outcome. In the studies which published the grading of TLS, all events were grades 3–5 (14, 19, 25, 26, 28). It is possible that the low rates of TLS are related to step-up dosing, or that pre-treatment with steroids and/or anti-CD20 monoclonal antibodies reduce the rate of TLS by debulking the tumor prior to bsAb exposure. As with other complications of bsAb use, factors which influence the development of TLS are likely to become clearer as larger studies are published. Clinicians should be aware that TLS is a possible complication and a risk-adapted approach to prevention, monitoring and management is recommended. TLS and infection rates are summarised in Table 5.

**TABLE 5:** Infection and TLS incidence.

Drug	Study	Disease	*n* =	Median follow-up	Infection, all grade	Infection, Gr 3–5	Febrile neutropenia, all grade	Tumor lysis syndrome, all grade
Glofitamab	Rentsch et al. (15)	DLBCL	9	246 d (15–482)	44%	ND	ND	11%
	Birtas Atesoglu et al. (16)	DLBCL	46	5.7 m (0.3–14.19)	ND	ND	ND	ND
	Sesques et al. (17)	B-NHL	63	DLBCL: 9.7 m (95% CI 8.1-11.8) Other*: ND	ND	27%	ND	ND
	Hutchings et al.^†^ (18)	B-NHL	35	ND	42.9%	ND	5.7%	ND
	Dickinson et al. (19)	DLBCL	154	12.6 m (0.1–22.1)	38%	ND	ND	1%
	Phillips et al. (20)	MCL	60	19.6 m (0–39)	73.3%	35%	ND	ND
	Haynes et al. (21)	NHL	142	6.1 m (IQR 2.5–9.1)	45%	ND	ND	ND
	Brooks et al. (22)	LBCL	70	5 m (ND)	ND	ND	ND	ND
	Tong et al. (23)	DLBCL/HGBL	28	3 m (ND)	ND	ND	ND	ND
Mosunetuzumab	Budde et al. (12)	FL	90	18.3 m (IQR 13.9–23.3)	20%	14%	0%	ND
	Bartlett et al. (24)	DLBCL	88	10.1 m (ND)	ND	12.5%	5.7%	0%
Epcoritamab	Thieblemont et al.* (13, 25)	LBCL	157	25.1 m (95% CI 24.0–26.0)	45.2%	14.6%	2.5%	1.3%
	Izutsu et al. (26)	B-NHL	9	14.9 (ND)	ND	ND	ND	11%
	Izutsu et al. (26)	DLBCL	36	8.4 m (95% CI 6.5–11.2)	44.4%	19.4%	2.8%	ND
	Haynes et al. (21)	NHL	38	6.5 m (IQR 1.5–8.5)	ND	ND	ND	ND
	Brooks et al. (22)	LBCL	139	5 m (ND)	ND	ND	ND	ND
Odronextamab	Bannerji et al. (14)	B-NHL	145	4.2 m (IQR 1.5–11.5)	49%	23%	ND	1%
	Ayyappan et al. (27)	DLBCL	141	26.2 m (ND)	ND	37%	ND	ND
	Kim et al. (28)	FL	128	20.1 (ND)	79.7%	42.2%	ND	0.8%

^†^Safety-evaluable (RP2D) cohort. *Data from initial dose expansion study and 2-year follow-up, published separately. ND, no data.

## Discussion

The use of bsAb therapy represents a major step forward in the management of B-cell NHL, with promising efficacy reported for both high-grade and low-grade lymphomas. Of note, real-world studies are emerging, and at this time only those for glofitamab and epcoritamab are available for review. In addition to their efficacy, they are readily available as an off-the-shelf product. While the toxicities are not insignificant, they are typically manageable and justifiable given the often unmet medical need, especially in the case of relapsed or refractory disease. Physicians utilizing these therapies should be well-versed in the management of toxicities, in particular the toxicities of CRS and ICANS unique to immune effector cell-engaging immunotherapies. Although both autologous and allogeneic CAR-T therapies are becoming more widespread, bsAbs will likely play an important part in the therapeutic landscape into the future.

In addition to understanding the management of established toxicities of T cell-engaging bsAbs, there are numerous strategies to prevent or minimize complications. Currently, bsAb therapy is predominantly initiated in the inpatient setting, although outpatient administration is under investigation (63). While patients with lower metabolic tumor volume (MTV) experienced lower rates of CRS and ICANS in several CAR-T studies, it is not known if this is also the case in bsAb therapy (64, 65). Nevertheless, patient factors including MTV as well as age, performance status, and prior lines of therapy, may impact outcomes. The use of step-up dosing, as well as pre-treatment debulking therapies, were established to mitigate CRS risk (18) but likely also reduce TLS risk. As experience with T cell-engaging bsAb therapies grows, it is probable that further optimisation of step-up dosing schedules will be possible, including adaptive strategies for patients experiencing toxicities. The use of appropriate infection prevention strategies is vital, including the use of prophylactic antibiotics and antivirals as per institutional policies. Clinicians should ensure that patients are up-to-date with age-appropriate vaccinations, and practice effective environmental risk mitigation strategies.

The choice of time-limited versus continuous therapy is likely to affect the toxicity profile. Glofitamab and mosunetuzumab have been studied with time-limited schedules, while odronextamab and epcoritamab have used continuous dosing until disease progression or unacceptable toxicity. While both methods show encouraging efficacy, the choice between time-limited and continuous treatment would ideally be investigated in a randomized control trial of the same agent. The evaluation of response-adapted strategies, such as ctDNA MRD, warrants additional study.

Further investigation into the pathogenesis and optimal treatment of CRS and ICANS when using bsAbs is warranted. An understanding of the risk factors for these toxicities may assist in developing individualized treatment strategies for patients. The observation that CRS was less frequently seen in patients who had received prior CAR-T cell therapy raises several questions worthy of further study. Possible explanations could be the development of resistance mechanisms within lymphoma cells, exhaustion of host T cells, prior exposure to lymphodepleting chemotherapy, or the induction of immunological tolerance to certain off-target effects. This was investigated by Crochet et al. (66) who found that response to prior bsAb therapy did not predict response to CAR-T. In addition, a history of CRS with a bsAb did not predict CRS with subsequent CAR-T.

Furthermore, in our experience, hypogammaglobulinaemia can occur following bsAb therapy. It is unclear to what extent this is due to the effect of the treatment itself versus being reflective of a heavily pre-treated, immunosuppressed patient population. This has not been sufficiently investigated as of yet and so the measurement and reporting of hypogammaglobulinaemia and the use of intravenous immunoglobulin would be of value in future studies.

The focus of this review has been on describing the toxicities of bsAbs in the setting of lymphoma monotherapy studies. Importantly, bsAbs are currently being evaluated in combination with other anti-lymphoma therapies both in the frontline (e.g., NCT04914741) and relapsed/refractory settings (e.g., NCT06508658), as well as in different lymphoma subtypes such as mantle cell lymphoma (e.g., NCT06084936). Safety and efficacy data from such studies, as well as from the use of these therapies in other clinical contexts such as post-CAR-T cell therapy and as maintenance therapies, is currently still awaited.
